# Class II and IV HDACs function as inhibitors of osteoclast differentiation

**DOI:** 10.1371/journal.pone.0185441

**Published:** 2017-09-27

**Authors:** Nicholas C. Blixt, Bora K. Faulkner, Kristina Astleford, Rosemary Lelich, Jacob Schering, Ekaterina Spencer, Rajaram Gopalakrishnan, Eric D. Jensen, Kim C. Mansky

**Affiliations:** 1 Departmment of Genetics, Cell Biology and Development, University of Minnesota, Minneapolis, Minnesota, United States of America; 2 Department of Developmental and Surgical Sciences, University of Minnesota, Minneapolis, Minnesota, United States of America; 3 Department of Diagnostic and Biological Sciences, University of Minnesota, Minneapolis, Minnesota, United States of America; Charles P. Darby Children's Research Institute, UNITED STATES

## Abstract

Histone deacetylases (HDACs) are negative regulators of transcription and have been shown to regulate specific changes in gene expression. In vertebrates, eighteen HDACs have thus far been identified and subdivided into four classes (I-IV). Key roles for several HDACs in bone development and biology have been elucidated through *in vitro* and *in vivo* models. By comparison, there is a paucity of data on the roles of individual HDACs in osteoclast formation and function. In this study, we investigated the gene expression patterns and the effects of suppressing individual class II (*Hdac4*, *5*, *6*, *9*, and *10*) and class IV (*Hdac11*) HDACs during osteoclast differentiation. We demonstrated that HDAC class II and IV members are differentially expressed during osteoclast differentiation. Additionally, individual shRNA-mediated suppression of *Hdac4*, *5*, *9*, *10* and *11* expression resulted in increased multinucleated osteoclast size and demineralization activity, with little to no change in the overall number of multinucleated osteoclasts formed compared with control shRNA-treated cells. We also detected increased expression of genes highly expressed in osteoclasts, including *c-Fos*, *Nfatc1*, *Dc-stamp* and *Cathepsin K*. These observations indicate that HDACs cooperatively regulate shared targets in a non-redundant manner.

## Introduction

Bone is a dynamic tissue that is constantly remodeled through degradation by osteoclasts and renewal by osteoblasts [1]. Proper coordination between these cell types is essential for maintaining structural integrity of the skeleton throughout development. Osteoclasts are large, multinucleated cells derived from hematopoietic stem cells/monocyte precursors [1]. Osteoclast differentiation is governed mainly by two important cytokines: macrophage colony stimulating factor (M-CSF) and receptor activator of nuclear factor kappa B ligand (RANKL) [2–5]. RANKL stimulates the expression of key transcription factors such as NF-κB, c-Fos, MITF, and NFATc1 [5–8] that are necessary for osteoclast differentiation and maturation. Osteoclasts are needed for normal bone functions such as bone remodeling and fracture repair. However, uncontrolled osteoclast activity can lead to skeletal disorders such as osteoporosis. Therefore, it is important to determine mechanisms that regulate transcription of osteoclast genes. This knowledge may reveal key modulators of bone resorption that can be considered as therapeutic targets.

The existence of tissue-specific transcription factors alone is inadequate to control temporal gene expression; co-factors are needed for chromatin remodeling and recruitment of RNA polymerase II. Histone acetyltransferases (HATs) and histone deacetylases (HDACs) control gene expression markings by being drafted to target genes through interactions with specific transcription factors [9]. Gene activation is linked to the recruitment of HATs by transcription factors. Conversely, HDACs bind to the same transcription factors and promote transcriptional repression. Thus, HATs and HDACs act as molecular switches for the functions of transcription factors. These processes have become essential mechanisms to examine regarding our understanding of bone physiology and diseases.

HDACs are negative regulators of transcription, and have been shown to induce specific changes to gene expression in various biological processes by deacetylating both histone and non-histone proteins [10–12]. Rooted in structural and functional similarities to yeast deacetylases, the 18 HDACs in the human genome are categorized into four classes: class I HDACs (HDAC1, 2, 3 and 8), class II HDACs (HDAC4, 5, 6, 7, 9 and 10), class III HDACs (Sirtuins1-7) and class IV HDACs (HDAC11) [13–19]. HDACs are emerging as important regulators of skeletal homeostasis [16]. Various *in vitro* studies have reported that HDAC inhibitors can repress osteoclast differentiation [20–22]. These inhibitors are broad-spectrum compounds that target multiple HDACs. However, the specific effects of individual HDACs on osteoclasts are largely unknown.

Previous work from our lab demonstrated that suppression of *Hdac*7 and *Hdac*3 have opposite effects on osteoclast differentiation *in vitro* [23]. *Hdac*7 suppression enhances differentiation, whereas suppression of *Hdac*3 inhibits osteoclast differentiation. Our lab [24] as well as Jin et al [25] demonstrated that HDAC7 inhibits osteoclastogenesis and bone resorption *in vivo*. We found that HDAC7 represses osteoclast differentiation through interacting with the transcription factor MITF [24], while Jin et al. showed that HDAC7 regulates NFATc1 activity [25]. Additionally, Jin et al [26] demonstrated that HDAC9 suppresses osteoclastogenesis through negatively regulating PPARγ. These findings suggest that each HDAC member can induce suppression of osteoclast differentiation through distinct and possibly multiple mechanisms. However, it is still unclear whether other HDAC members play separate and pivotal roles in osteoclastogenesis.

The goal of this study was to investigate the functions of class II and IV HDACs during osteoclastogenesis and to determine whether any redundant roles exist for the class II and IV HDACs. Our results revealed that suppression of individual HDACs enhance osteoclast differentiation, potentially through repression of different proteins within the same cellular pathways. We anticipate that characterization of changes in gene expression patterns due to specific suppression of each HDAC in osteoclasts will further our understanding of how class II and IV HDACs regulate osteoclastogenesis. This knowledge will lead to potential new therapies in treating resorption-mediated bone diseases.

## Experimental procedures

### Ethics

The use and care of the mice was reviewed and approved by the University of Minnesota Institutional Animal Care and Use Committee, IACUC protocol number 150732820A. Euthanasia was performed by CO_2_ inhalation. Isolation and culture of osteoclasts from mouse bone marrow cells, as well as virus generation and viral transduction of osteoclasts were performed under approval of the University of Minnesota Institutional Biosafety Committee, permit number 1506-32712H.

### Primary osteoclast cell culture

Primary bone marrow macrophages (BMMs) were isolated from the femora and tibiae of C57BL/6 mice. The femora and tibiae were dissected out and adherent tissue was removed. The epiphyses of these long bones were removed, and the bone marrow was flushed from the diaphysis. Red blood cells were lysed from the flushed marrow using red blood cell lysis buffer (150 mM NH_4_Cl, 10 mM KHCO_3_, 0.1 mM EDTA, pH 7.4), and the resulting cells were plated and cultured overnight in 100 mm tissue culture dishes (MidSci) in osteoclast media (phenol red-free alpha-MEM [Gibco] with 5% fetal bovine serum [Hyclone], 25 units/mL penicillin/streptomycin [Invitrogen], 400 mM L-Glutamine [Invitrogen], and supplemented with 1% CMG 14–12 culture supernatant containing M-CSF). CMG 14–12 cell line was obtained from Dr. Sunao Takeshita (Nagoya City University, Nagoya, Japan) [27]. The non-adherent cell population was then re-plated in 12-well plates (MidSci) at 2 x 10^5^ cells/well in osteoclast media supplemented with 1% CMG 14–12 culture supernatant for 48 hours. Cells were then fed every two days with osteoclast media containing 1% CMG culture supernatant plus 10 ng/mL RANKL (R&D Systems) to stimulate osteoclastogenesis.

### Tartrate resistant acid phosphatase (TRAP) staining

After five days of culture with 1% CMG 14–12 culture supernatant and 10 ng/ml RANKL, primary osteoclasts were rinsed in PBS and fixed with 4% paraformaldehyde for 20 minutes. Cells expressing TRAP were stained with Naphthol AS-MX phosphate and Fast Violet LB salt protocol as previously described [24]. The stained cells were then imaged and photographed with light microscopy and analyzed using NIH ImageJ to measure the number and size of TRAP-positive multinuclear cells.

### Demineralization assay

BMMs were plated on calcium phosphate-coated plates (Corning) and cultured as above. After five days of stimulation with 1% CMG 14–12 culture supernatant and 10 ng/ml RANKL, plates were processed according to the manufacturer’s instructions, the demineralized area was photographed by dark field microscopy and analyzed using NIH ImageJ.

### Lentiviral infection of osteoclasts

Lentiviral vectors (Open Biosystems) encoding shRNAs against *Hdac4* (TRCN0000039249 and TRCN0000039252), *Hdac5* (V2LMM 72835 and V3LMM 432047), *Hdac6* (V2LMM 61798 and V2LMM 79557), *Hdac9* (V3LMM 481592 and V3LMM 481594), *Hdac10* (V3LMM 425386 and V3LMM 425387), *Hdac11* (V2LMM 24029 and TRCN0000039224), or a control shRNA were used to produce replication-defective lentivirus according to the manufacturer’s protocols. Viral stocks were titrated by infection in HeLa cells. BMMs were isolated and cultured as described above. 48 hours after seeding the non-adherent population, lentiviruses were added at 10 MOI and incubated for 18 hours at 37°C in the presence of 1% CMG 14–12 culture supernatant. The following day cultures were stimulated with 1% CMG 14–12 culture supernatant and 10 ng/ml RANKL. Cells were used for either RNA extraction after 48 hours with 1% CMG 14–12 culture supernatant and RANKL treatment, or TRAP staining or demineralization assays after 5 days with 1% CMG 14–12 culture supernatant and RANKL.

### RNA isolation and real-time PCR

RNA was harvested from cells plated in triplicate using TRIZOL Reagent (Ambion, Life Technologies) and quantified using UV spectroscopy. cDNA was then prepared from 1 μg of purified RNA using iScript cDNA Synthesis Kit (Bio-Rad) per the manufacture’s protocol. Quantitative real-time PCR (qRT-PCR) was performed in duplicate using CFX Connect Real-Time PCR system (Bio-Rad). Each 20 μl reaction mixture contained 1 μl cDNA, 10 μL iTaq Universal Sybr Green Supermix, and 500 nM forward and reverse primers. The PCR conditions were as follows: 95°C for 3 minute, and 40 cycles of 94°C for 15 seconds, 56°C for 30 seconds, and 72°C for 30 seconds, followed by melt curve analysis (95°C for 5 seconds, 65°C for 5 seconds, and then 65°C to 95°C with 0.5°C increase for 5 seconds). Experimental genes were normalized to *Hprt*. Primers amplified with equal efficiencies. Their sequences used are listed in S1 Table. All measurements were analyzed using the ΔΔCT method.

### Immunoblot analysis

Protein cell lysates were harvested from primary osteoclasts in modified RIPA buffer (50 mM Tris pH 7.4, 150 mM NaCl, 1% IGEPAL, 0.25% sodium deoxycholate, 1 mM EDTA) supplemented with Halt Protease & Phosphatase Inhibitor Cocktail (Thermo Scientific). Lysates were cleared by centrifugation. Proteins were resolved by SDS-PAGE, transferred to PVDF membrane (Millipore), blocked, and blotted in primary antibody overnight at 4°C. The next day, blots were incubated with horseradish peroxidase conjugated secondary antibody (G.E. Healthcare) for 1 hour at room temperature. Antibody binding was detected using western blotting detection kit (Western Bright Quantum, Advansta). The following primary antibodies were all used at 1:1000 dilution: polyclonal HDAC4 produced using peptide corresponding to amino acids 14–28 of human HDAC4 –Sigma Aldrich (H9536, Antibody ID# AB477079); monoclonal HDAC5 produced using peptide corresponding to amino acids 371–443 of human HDAC5—Santa Cruz Biotechnology (SC-133225, Antibody ID# 2116791); polyclonal HDAC9 produced using peptide corresponding to amino acids 112–129 of HDAC9 –Thermo Scientific (PA5-23346, Antibody ID# AB2540870); polyclonal MITR produced from peptide against human HDAC9—Sigma Aldrich (SAB4503694, Antibody ID# AB10751345); polyclonal HDAC10 produced using peptide corresponding to amino acids 2–16 of human HDAC10 –Sigma Aldrich (H3413, Antibody ID# AB261940); polyclonal HDAC11 produced using peptide corresponding to amino acids 2–16 of human HDAC11 –Sigma Aldrich (H4539, Antibody ID# AB532246) and polyclonal ACTIN-Santa Cruz Biotechnology produced using carboxy terminus of human actin (SC-1616, Antibody ID# AB630836). The secondary antibodies used were all used at 1:10,000 dilution: anti-rabbit (NA-934, Antibody ID# AB772206) and anti-goat (SC-2020, Antibody ID# AB631728). All densitometry data was generated using the Image Lab Software (Bio Rad) following the manufacturer’s instructions for normalizing data to a housekeeping protein. The calculated volume intensity for HDAC expression was normalized to its corresponding actin from the same membrane.

### Statistical analysis

All experiments were run in triplicate, performed three times, and results are expressed as mean ± standard deviation. Student’s t-test or one-way ANOVA analysis followed by a Tukey’s multiple comparison test were used to compare data using Graph-Pad Prism version 7.

## Results

### Expression of class II and IV HDACs during osteoclastogenesis

**Fig 1:**
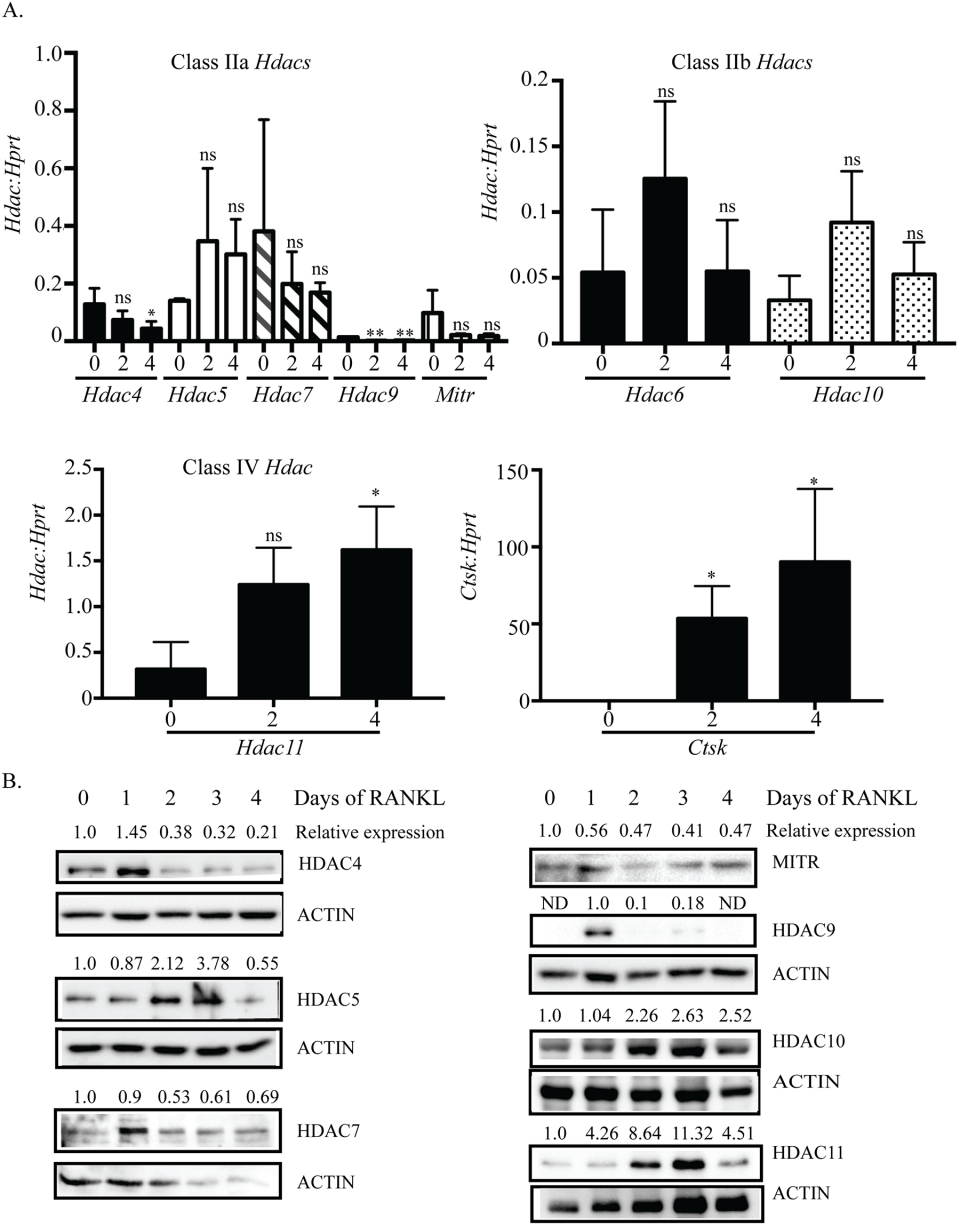
Class II and IV *Hdac* expression during osteoclast differentiation. BMM cells from C57BL/6 mice were cultured in M-CSF only (day zero) or in M-CSF plus RANKL (day two and day four) to stimulate osteoclast differentiation. Class II and IV *Hdac* RNA expression (A) on day zero, day two and day four of osteoclast differentiation. qRT-PCR data shown are the mean of three independent experiments. * p <0.05 comparing vs. day zero, ns = not significant. Representative protein expression (B) of class II and IV HDACs on each day of osteoclast differentiation. Displayed relative expression values are HDAC expression relative to the normalized expression on day zero as calculated by Image Lab Software (BioRad). In the case of HDAC9 no band was identified for day zero so data is expressed relative to day one. The same lysates were analyzed for HDAC9 and MITR expression and therefore, have the same actin loading control.

To investigate the roles of class II and IV HDACs during osteoclastogenesis we first examined their expression through the process of osteoclast differentiation. Non-adherent cells from C57BL/6 (wild-type) mouse bone marrow cells were cultured with 1% CMG for two days to promote bone marrow macrophages (BMMs) proliferation, and then treated with M-CSF plus RANKL for four more days to stimulate osteoclast differentiation. All *Hdac* members in class IIa, IIb and IV were expressed in both proliferating BMMs (day zero with M-CSF treatment) and differentiating osteoclasts (day one-day four with M-CSF plus RANKL treatment) (Fig 1A). Among these HDACs, the expression of *Hdac4*, *Hdac7*, *Hdac9* and Histone deacetylase related protein (*Hdrp/Mitr)* were significantly down-regulated upon RANKL stimulation (day zero vs day two) and expression remained low during osteoclast differentiation (day one-day four, S1 Fig). Conversely, *Hdac5*, *Hdac6*, *Hdac10* and *Hdac11* expression increased with RANKL expression (Fig 1A, day zero vs day two). Jin et al [26] reported similar trends in HDAC mRNA expression. *Hdac11*, *Hdac5* and *Hdac7* were the most highly expressed *Hdac* RNAs during osteoclast differentiation. As expected, the expression of the osteoclast marker gene Cathepsin K (*Ctsk*) was significantly increased upon RANKL stimulation and served as a positive control for osteoclast differentiation (Fig 1A, bottom right panel). HDAC protein expression as measured by western blot demonstrated similar expression patterns to the mRNAs (Fig 1B, S2 Fig). These results show that HDAC class II and IV members are differentially expressed during osteoclast differentiation.

### Effect of shRNA suppression of *Hdac*s on osteoclast differentiation

Since we detected expression of all the examined *Hdacs* in osteoclast cultures, we next asked how suppression of each individual *Hdac* would affect osteoclast formation or function. To this end, we used lentiviral vectors encoding shRNAs against these *Hdacs*. Prior to beginning RANKL stimulation, BMMs from wild-type mice were infected with one of two lentiviral vectors encoding an shRNA against an *Hdac* or a control shRNA. For each *Hdac*, we obtained similar results with both shRNAs indicating that the outcomes were not due to off-target effects. Following infection with shRNAs, we confirmed target gene knockdown by qRT-PCR and examined changes in the expression of *c-Fos*, *Nfatc1*, *Dc-stamp* and *Ctsk* genes important for osteoclast formation or function. Because our experiments produced similar results in our analysis of osteoclast differentiation and activity assays using the two different *Hdac* shRNA, we grouped our PCR data together from the *Hdac* shRNA experiments for qRT-PCR. We employed TRAP staining and demineralization assays to measure osteoclast differentiation, fusion and activity.

### Suppression of *Hdac4* increases osteoclastogenesis

**Fig 2:**
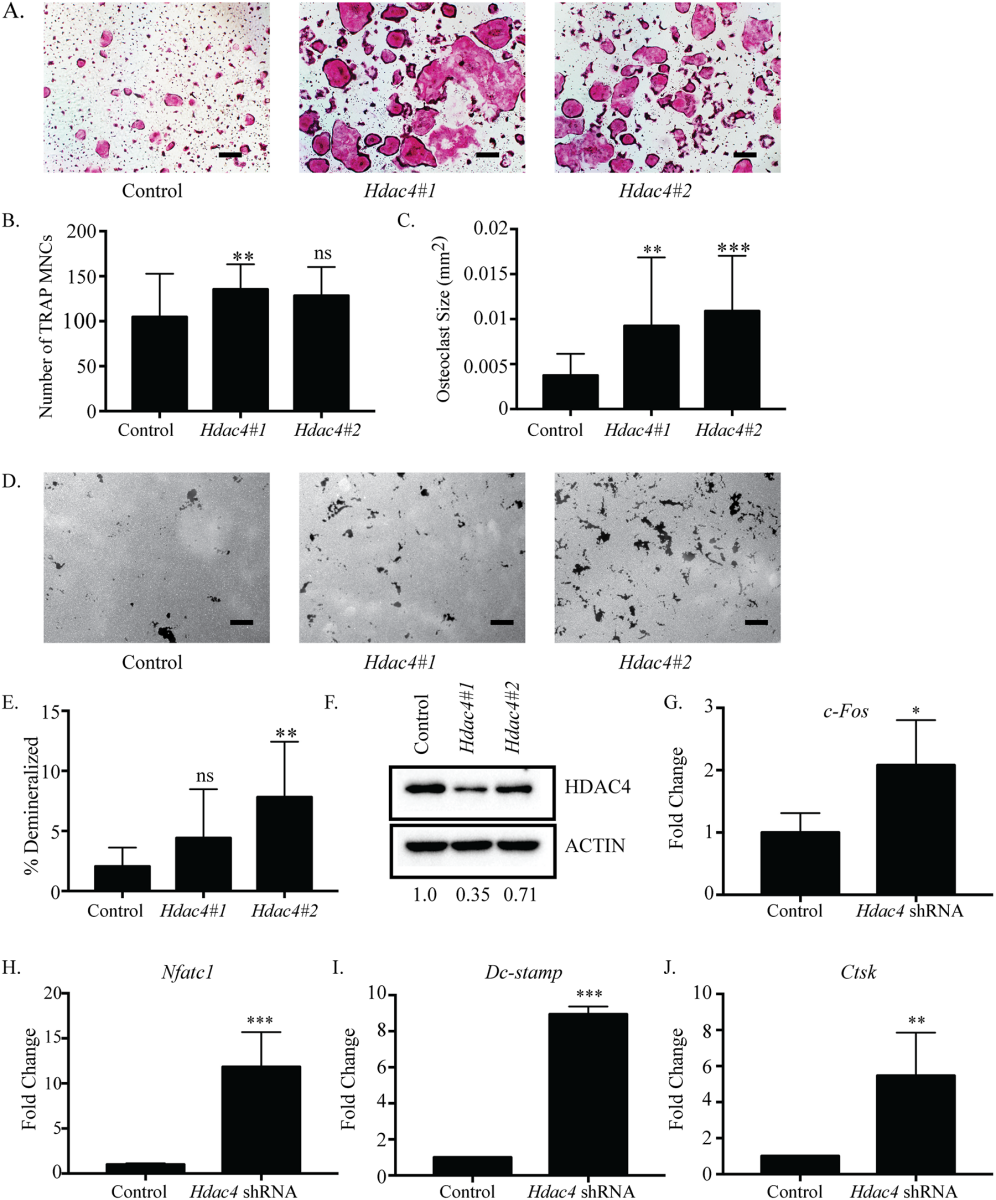
Accelerated osteoclast differentiation in *Hdac4*-suppressed osteoclasts. Representative images of TRAP staining (A) of osteoclast cultures infected with control or *Hdac4* shRNA-expressing lentivirus. *Hdac4*#1 represents *Hdac4* shRNA #1and *Hdac4*#2 represents *Hdac4* shRNA #2. Number (B) and size (C) of TRAP-positive MNCs. Representative images (D) and quantification (E) of demineralization activity of control and *Hdac4* shRNA-expressing osteoclast cultures grown on calcium phosphate-coated plates. Scale bar represents 200 μm. Western blot (F) of control and *Hdac4* shRNA-expressing cells with relative expression of shRNA expressing cells relative to control expressing cells indicated under the blots. Expression profile (G-J) of osteoclast genes *c-Fos*, *Nfatc1*, *Dc-stamp and Ctsk*. Data presented are the mean of three independent experiments. * p <0.05; ** p <0.01; *** p <0.001; ns = not significant compared to control infected cells.

First, we investigated the effects of silencing *Hdac4* on osteoclastogenesis. Cells infected with *Hdac4* shRNA #1 or #2 showed enhanced osteoclast differentiation compared to control shRNA (Fig 2A). The observed TRAP-positive multinuclear cells (MNCs) from both *Hdac4*-shRNA cultures were more numerous, and their size significantly increased (3-fold) relative to the control (Fig 2B and 2C). Demineralization assays on calcium phosphate-coated plates demonstrated cells infected with *Hdac4* shRNA #2 produced significantly increased total number of pits, average pit size, and total percent demineralized area compared to control shRNA infected cells. *Hdac4* shRNA #1 infected cells showed similar trends (Fig 2D and 2E, S3 Fig). Moreover, BMMs infected with either *Hdac4* shRNA showed *Hdac4* RNA expression levels reduced by approximately 50% (S3 Fig), and *Hdac4* protein expression levels reduced between 30–65% (Fig 2F). *Hdac4* shRNA increased expression of *c-Fos*, *Nfatc1*, *Dc-stamp* and *Ctsk* compared to control shRNA (Fig 2G–2J). Taken together these results reveal that *Hdac4* suppression increased osteoclast differentiation by producing larger and more numerous osteoclasts, suggesting HDAC4 inhibits osteoclastogenesis.

### *Hdac5* is a negative regulator of osteoclastogenesis

**Fig 3:**
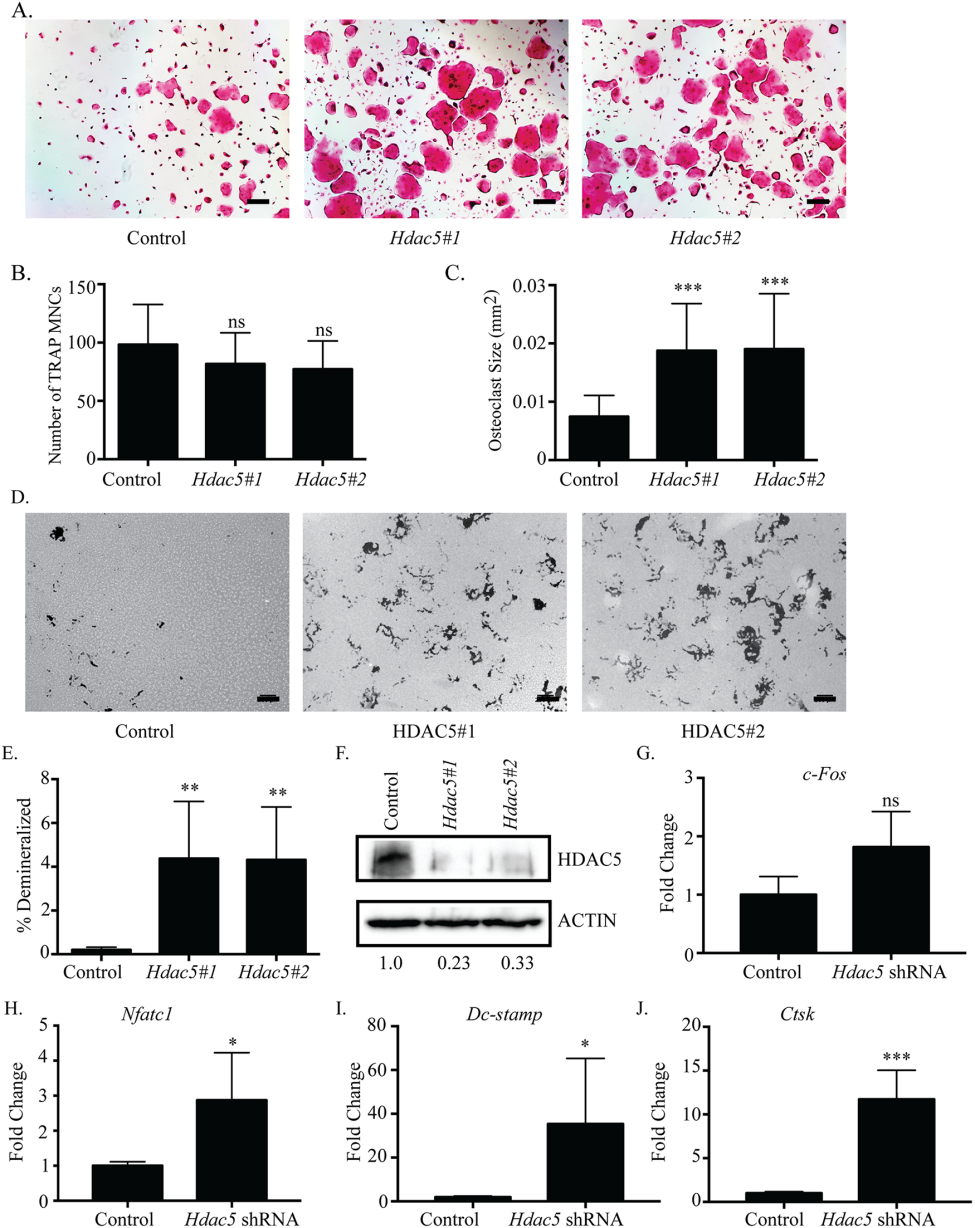
Suppression of *Hdac5* enhances osteoclast differentiation. Representative images of TRAP staining (A) of osteoclast cultures. *Hdac5*#1 represents *Hdac5* shRNA #1and *Hdac5*#2 represents *Hdac5* shRNA #2. Quantification of the number (B) and average size of TRAP-stained multinucleated osteoclasts (C). Representative photographs (D) and quantification (E) of demineralization activity of control and *Hdac5* shRNA-expressing osteoclast cultures grown on calcium phosphate-coated plates. Scale bar represents 200 μm. Western blot (F) of control and *Hdac5* shRNA-expressing cells with relative expression of shRNA expressing cells relative to control expressing cells indicated under the blots. Expression profile (G-J) of osteoclast genes *c-Fos*, *Nfatc1*, *Dc-stamp and Ctsk*. Data presented are the mean of three independent experiments. * p <0.05; ** p <0.01; *** p < 0.001; ns = not significant compared to control infected cells.

We investigated the effects of suppressing expression of the Class IIa deacetylase *Hdac5*. Osteoclast differentiation in either *Hdac5*-shRNA culture was enhanced compared to control shRNA (Fig 3A). The average size of TRAP-positive MNCs in *Hdac5*-shRNA cells was significantly increased (Fig 3B). However, the number of TRAP-positive MNCs per well in *Hdac5*-shRNA cells did not significantly change compared to control shRNA (Fig 3C). When osteoclast activity was determined, cells infected with each *Hdac5*-shRNA had significantly increased total number of pits, average pit size, and total percent demineralized area (Fig 3D and 3E, S4 Fig). The cultured osteoclasts also showed reduced *Hdac5* mRNA expression levels (S4 Fig), and approximately 70–80% reduction in HDAC5 expression as measured by western blot (Fig 3F). *Hdac5*-suppressed osteoclasts showed an upward but not significant change in expression of *c-Fos* (Fig 3G); while expression of genes important for osteoclast formation or function including *Nfatc1*, *Dc-stamp*, and *Ctsk* (Fig 3H–3J) were significantly increased. These findings indicate that *Hdac5* suppression enhances osteoclast differentiation through increased expression of genes such as *Nfatc1*, *Dc-stamp*, and *Ctsk* but independent of changes to *c-Fos*.

### *Hdac6* suppression does not affect osteoclast differentiation

**Fig 4:**
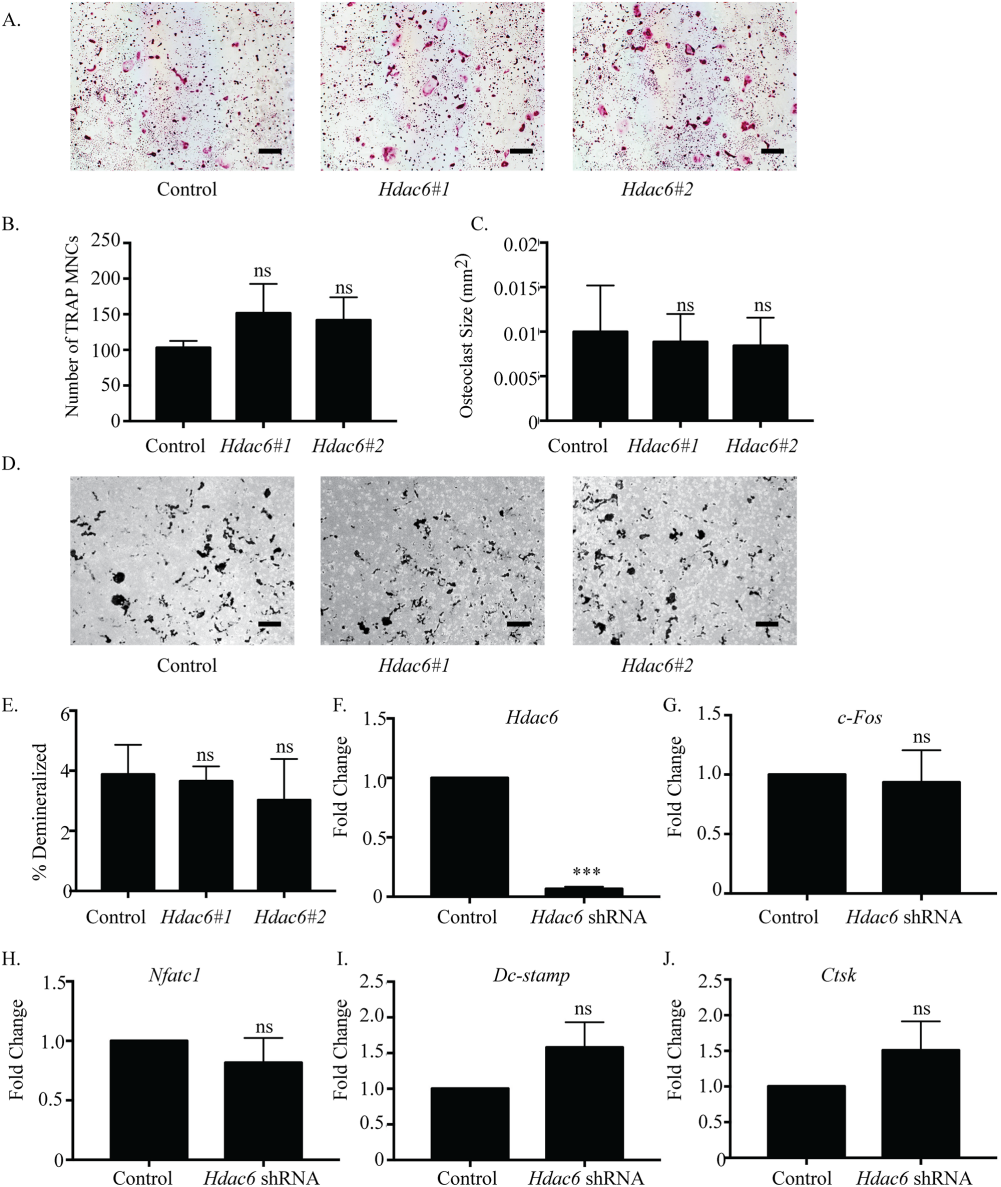
*Hdac6* suppression does not affect osteoclast differentiation. Representative images of TRAP staining (A) of osteoclast cultures. *Hdac6*#1 represents *Hdac6* shRNA #1and *Hdac6*#2 represents *Hdac6* shRNA #2. Quantification of (B) number and (C) size of TRAP-positive MNCs. Representative photographs (D) and quantification (E) of demineralization activity of control and *Hdac6* shRNA-expressing osteoclast cultures grown on calcium phosphate-coated plates. Scale bar represents 200 μm. qRT-PCR (F) of control and *Hdac6* shRNA-expressing cells. Expression profile (G-J) of *c-Fos*, *Nfatc1*, *Dc-stamp*, *and Ctsk*. Data presented are the mean of three independent experiments. *** p < 0.001; ns = not significant compared to control infected cells.

HDAC6, a member of class IIb HDAC family and tubulin deacetylase, has been shown to regulate tubulin dynamics and stability of the podosome belt in mature osteoclasts [28, 29]. However, the role of HDAC6 in early stages of osteoclast differentiation is unknown. There was no significant change in the number or size of TRAP-positive MNCs with *Hdac6* suppression (Fig 4A–4C). Moreover, *Hdac6*-shRNA #1 showed no significant changes in the demineralization assay (Fig 4D and 4E, S5 Fig), but *Hdac6*-shRNA #2 had a significant decrease only in the average pit size (S4 Fig). Commercially verifiable antibodies to HDAC6 are not available; therefore, we verified both *Hdac6* shRNAs significantly repressed *Hdac6* expression by qPCR (Fig 4F). However, *c-Fos*, *Nfatc1*, *Dc-stamp*, and *Ctsk* were not significantly changed by *Hdac6* suppression (Fig 4G–4J). These data show that knockdown of HDAC6 does not have any impact on osteoclast differentiation, at least within the parameters and genes analyzed.

### Suppression of *Hdac*9 inhibits osteoclastogenesis

**Fig 5:**
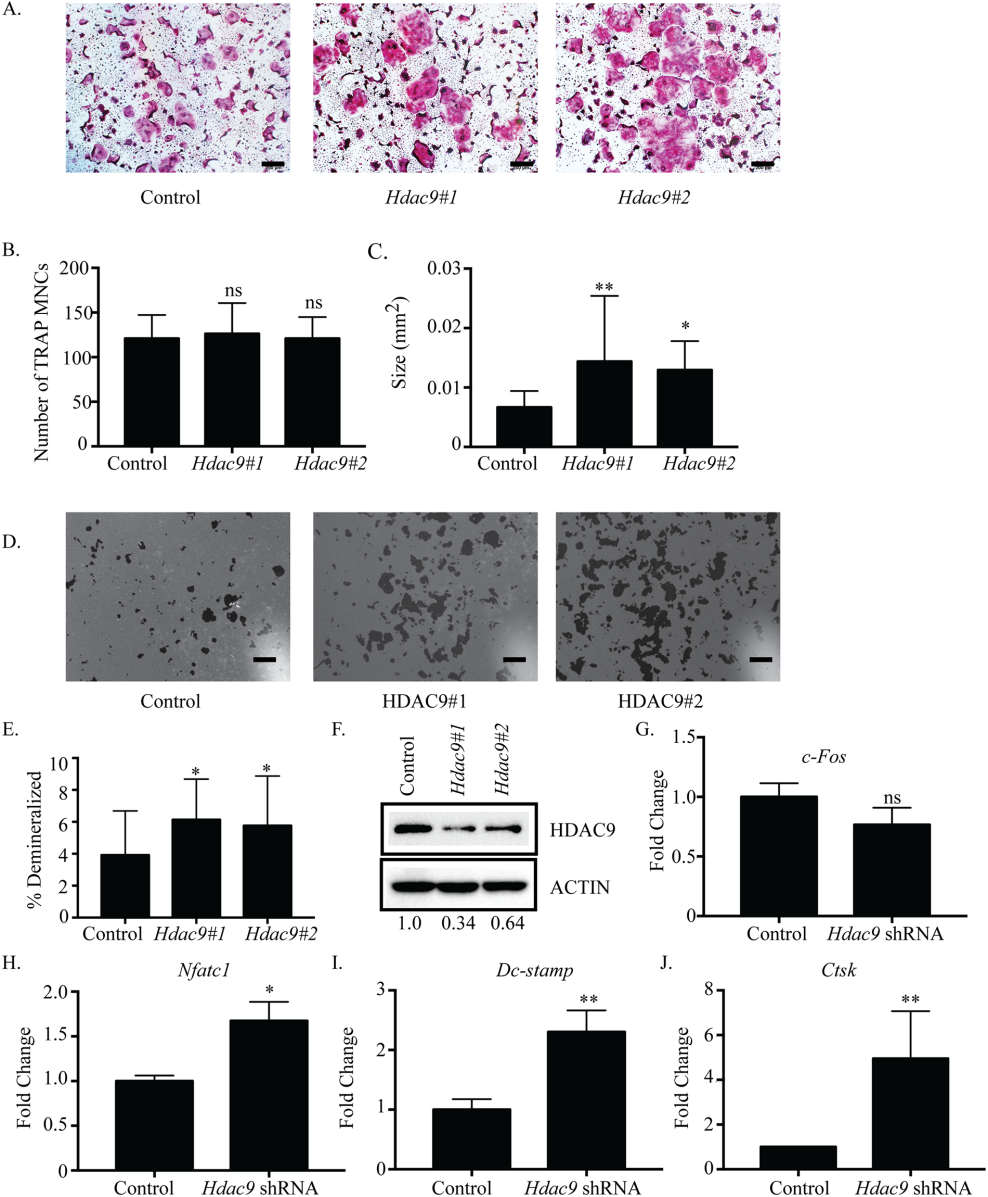
Suppression of *Hdac9* inhibits osteoclast differentiation. Representative images of TRAP staining (A) of osteoclast cultures infected with control or *Hdac9* shRNA-expressing lentiviruses. *Hdac9*#1 represents *Hdac9* shRNA #1and *Hdac9*#2 represents *Hdac9* shRNA #2. Number (B) and size (C) of TRAP-positive MNCs. Representative photographs (D) and quantification (E) of demineralization activity of control and *Hdac9* shRNA-expressing osteoclast cultures grown on calcium phosphate-coated plates. Scale bar represents 200 μm. Western blot (F) of control and *Hdac9* shRNA-expressing cells with relative expression of shRNA expressing cells relative to control expressing cells indicated under the blots. Expression profile (G-J) of *c-Fos*, *Nfatc1*, *Dc-stamp and Ctsk*. Data presented are the mean of three independent experiments. * p <0.05; ** p <0.01; ns = not significant compared to control infected cells.

We next used shRNAs to characterize the role of *Hdac9 and Mitr* in osteoclasts. Although our gene expression data (Fig 1) indicate that MITR is the predominant form of HDAC9 in osteoclasts, we were unable to identify shRNAs that distinguish between *Mitr* and full-length *Hdac9*. Consequently, both shRNAs used are predicted to target *Hdac9* and *Mitr*. Suppression of *Hdac9* increased the size of TRAP-positive MNCs, but had little effect on the number of osteoclasts formed (Fig 5A–5C). Demineralization assays showed that in *Hdac9*-shRNA infected cells, average pit size and demineralized area were significantly increased (Fig 5D and 5E, S6 Fig). As expected, in *Hdac9*-suppressed cells there was a reduction in *Hdac9* protein expression (Fig 5F) as well as *Hdac9* RNA (S6 Fig). *Hdac9*-suppressed cells revealed a slight but not significant reduction in *c-Fos* expression (Fig 5G), while expression of *Nfatc1*, *Dc-stamp*, and *Ctsk* was increased compared with control (Fig 5H–5J). These observations indicate that HDAC9/MITR inhibits osteoclastogenesis.

### HDAC10 activity inhibits osteoclastogenesis

**Fig 6:**
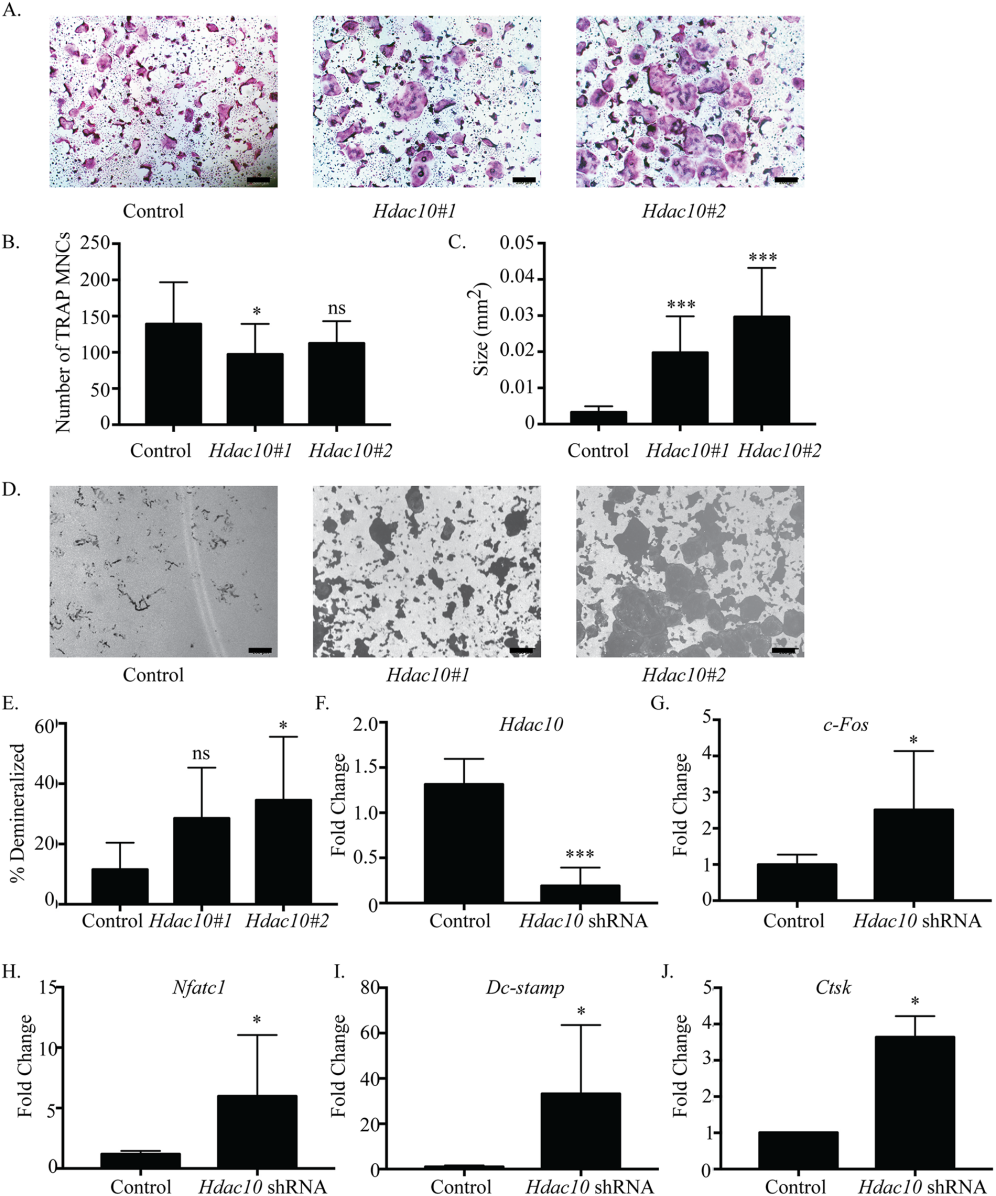
*Hdac10* suppression accelerates osteoclast differentiation. Representative images of TRAP staining (A) of osteoclast cultures. *Hdac10*#1 represents *Hdac10* shRNA #1and *Hdac10*#2 represents *Hdac10* shRNA #2. Quantification of number (B) and size (C) of TRAP-positive MNCs. Representative images (D) and quantification (E) of demineralization activity of control and *Hdac10* shRNA-expressing osteoclast cultures grow on calcium phosphate-coated plates. Scale bar represents 200 μm. qRT-PCR (F) of control and *Hdac10* shRNA-expressing cells. Expression profile (G, H, I and J) of *c-Fos*, *Nfatc1*, *Dc-stamp*, *and Ctsk*. Data presented are the mean of three independent experiments. * p < 0.05; *** p <0.001; ns = not significant compared to control infected cells.

HDAC6 and HDAC10 are members of class IIb HDAC family. HDAC6 functions as a tubulin deacetylase, and its role as a disrupter of the actin belt in mature osteoclasts has been well established [30, 31], whereas the biological function(s) of HDAC10 remain largely unknown [32, 33]. To explore HDAC10’s role in osteoclast differentiation, we examined the effects of suppressing its expression (Fig 6A), finding that in *Hdac10*-suppressed cells showed a decrease in the mean number of TRAP-positive MNCs per well (Fig 6B). The average size of TRAP-positive MNCs in *Hdac10*-suppressed cells was significantly increased (Fig 6C). Demineralization assays show total number of pits, average pit size and percent demineralized area was increased with knockdown of *Hdac10* (Fig 6D and 6E, S7 Fig). As expected, *Hdac10*-suppressed osteoclasts showed a reduction in *Hdac10* mRNA expression compared to control shRNA (Fig 6F). We were unable to obtain a reliable western blot verifying HDAC10 protein reduction in shRNA expressing cells due to technical issues. Expression of *c-Fos*, *Nfatc1*, *Dc-stamp*, and *Ctsk* was significantly increased (Fig 6G–6J). These data indicate that reduction of *Hdac10* expression enhanced osteoclast formation, suggesting that HDAC10, unlike HDAC6, may negatively regulate osteoclast differentiation.

### HDAC11 inhibits osteoclastogenesis

**Fig 7:**
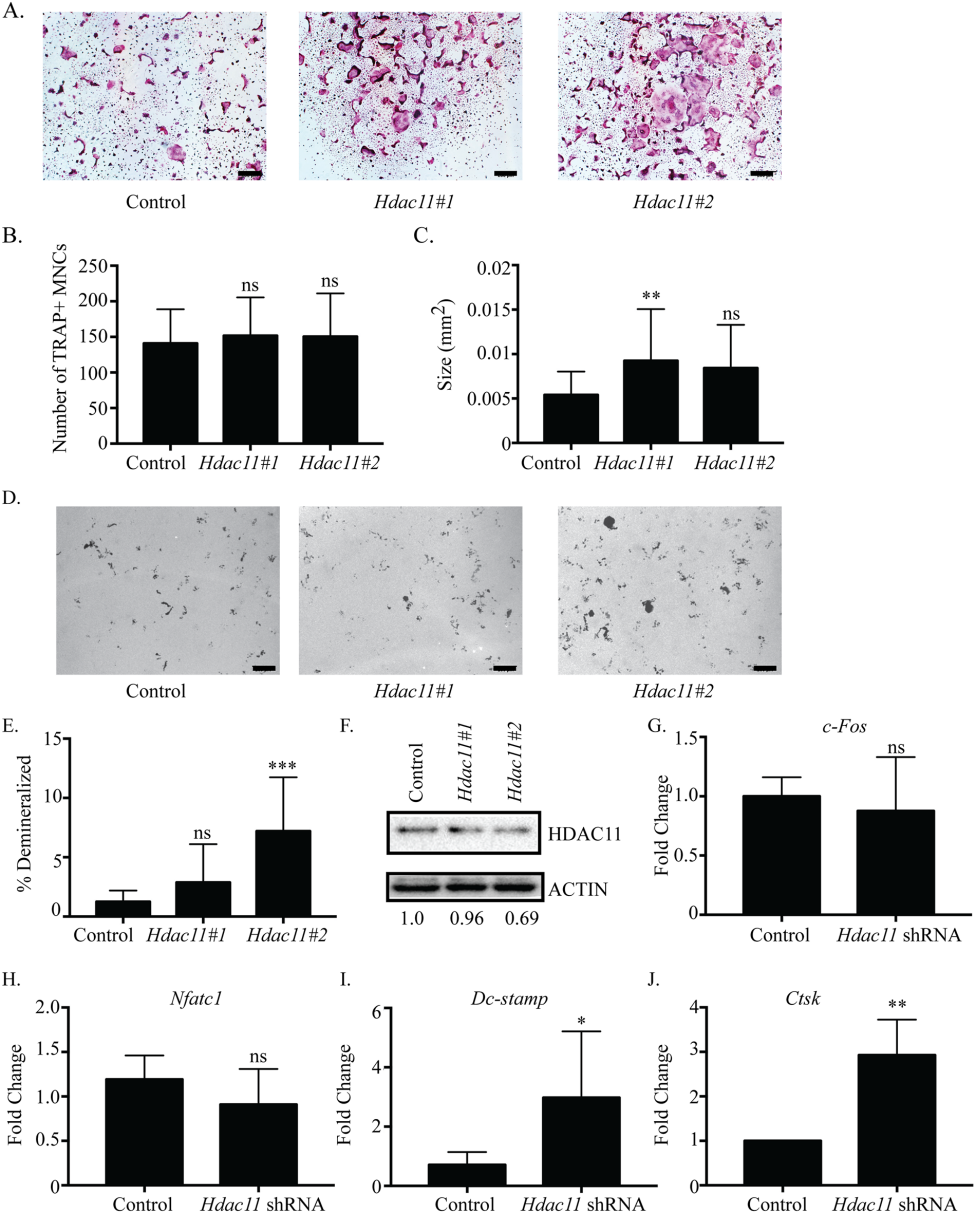
Suppression of *Hdac11* inhibits osteoclast differentiation. Representative images of TRAP staining (A) of osteoclast cultures. *Hdac11*#1 represents *Hdac11* shRNA #1and *Hdac11*#2 represents *Hdac11* shRNA #2. Histomorphometric analysis of TRAP-stained osteoclasts (B-C). Representative photographs (D) and quantification (E) of demineralization activity of control and *Hdac11* shRNA-expressing osteoclast cultures grown on calcium phosphate-coated plates. Scale bar represents 200 μm. Western blot (F) of control and *Hdac11* shRNA-expressing cells with relative expression of shRNA expressing cells relative to control expressing cells indicated under the blots. Expression profile (G, H, I and J) of *c-Fos*, *Nfatc1*, *Dc-stamp*, *and Ctsk*. Data presented are the mean of three independent experiments. * p < 0.05; ** p <0.01; *** p <0.001; ns = not significant compared to control infected cells.

We examined the effects of suppressing *Hdac11* on osteoclast differentiation. Cells infected with either *Hdac11* shRNA had enhanced osteoclast differentiation (Fig 7A). Quantitative analysis of these cultures indicated that there was no significant difference in the average number of TRAP-positive MNCs formed in either *Hdac11* shRNA cultures compared with control shRNA (Fig 7B). However, the average size of TRAP-positive MNCs was increased in both *Hdac11* shRNA cultures (Fig 6C). *Hdac11* shRNA #2 in demineralization assays increased the total number of pits, average pit size and total percent demineralized area significantly (Fig 7D and 7E, S7 Fig). *Hdac11* shRNA #1 infected cells did not show a significant difference in average pit size but did show a difference in the total number of pits and total percent demineralized area (Fig 7D and 7E, S8 Fig). The *Hdac11* shRNAs moderately reduced *Hdac11* RNA (S8 Fig) and protein expression (Fig 7F), which led to increased *Dc-stamp* and *Ctsk* expression (Fig 7I and 7J) but no change in *c-Fos* (Fig 7G) and *Nfatc1* expression (Fig 7H). These results reveal that *Hdac11* suppression enhanced osteoclast formation, thus suggesting *Hdac11* acts as an inhibitor of osteoclast differentiation.

## Discussion

To better understand how osteoclast differentiation and activity are regulated, it is necessary to investigate classes of transcriptional activators and repressors that function in osteoclasts. HDACs are a well-known class of transcriptional repressors that have been shown to be active in osteoclasts. Due to their high sequence homology and interactions with the same proteins in other cell types [14, 34], some HDACs may perform redundant roles in regulating osteoclast differentiation. However, recent studies demonstrated two class II HDACs, HDAC7 and HDAC9, repressed osteoclast differentiation using unique mechanisms. Deletion of either *Hdac7* or *Hdac9* in BMMs resulted in enhanced osteoclast differentiation *in vitro*, and increased bone resorption and osteopenia in mice *in vivo* [24, 26]. Our lab concluded HDAC7 interacted with and repressed the activity of MITF [24], another group demonstrated via reporter assays that HDAC7 might repress NFATc1 activity [25], and HDAC9 repressed PPAR-γ [26]. Though these mechanisms are different, it was still possible that the remaining Class II HDACs performed redundant roles in osteoclasts, in which case we hypothesized that loss of any one HDAC would be compensated for by the remaining proteins. To address this issue, we analyzed mRNA and protein expression, and the effects of shRNA-mediated knockdown of most class II (HDAC4, 5, 6, 9, and 10) and class IV (HDAC11) HDACs during osteoclast differentiation. To help identify how each HDAC was affecting osteoclasts, we used TRAP staining, demineralization assays, and qRT-PCR to evaluate changes in differentiation, activity, or gene expression due to *Hdac* knockdown. Individual knockdown of *Hdac4*, *5*, *9*, *10*, and *11* produced similar phenotypes, which was observed as increased size of multinuclear TRAP-positive MNCs and percent demineralization of a calcium phosphate matrix, but little to no change in multinuclear TRAP-positive osteoclast number (Figs 2, 3 and 5–7). Additionally, temporal expression patterns of these HDACs were also different from each other, which is in agreement with a previous study [26]. The presence of similar osteoclast phenotypes when different HDACs are knocked down and their distinct expression profiles suggests that most HDACs have at least some unique targets rather than fully redundant roles.

### Inhibiting differentiation in early stages of osteoclast differentiation

*Hdac9* and the related *Mitr* are highly expressed in BMMs but drastically decrease in expression after RANKL stimulation. *Hdac4* follows a similar trend but maintains a low level of expression throughout differentiation (Fig 1A and 1B). The initially high levels of HDAC9 and HDAC4 in BMMs which begin decreasing with the onset of RANKL stimulation could indicate that the presence of these HDACs is associated with maintaining proliferation rather than differentiation. Knockdown of *Hdac4* showed a small but significant increase in osteoclast number (Fig 2B) and a trend towards increased *c-Fos* expression (Fig 2G). Supporting this, osteoclasts from *Hdac4*-KO mice showed increased *c-Fos* expression (data not shown, manuscript in preparation). Knockdown of *Hdac9* demonstrated increased differentiation marker expression and osteoclast size but not increased osteoclast number or *c-Fos* expression, indicating HDAC9 may not be involved in proliferation through regulating *c-Fos* expression (Fig 5, [26]. Previous research on HDAC9 in osteoclasts showed both increased cell number and size of osteoclasts from global *Hdac9*-KO mice [26]. These contrasting results could be explained by the global deletion of *Hdac9* affecting the BMM population at a stage before our transient shRNA studies began. Considering this, both previous and our work show upregulated expression of genes important for differentiation, implying knockdown of *Hdac9* promotes a switch from proliferation to differentiation earlier than normal (Fig 5).

MITR is a truncated splice variant of HDAC9 that displays co-repressor activity despite lacking the C-terminal deacetylase catalytic domain. Specific suppression of *Mitr* but not of *Hdac9* expression in neurons leads to cell death, demonstrating a necessary role for MITR separate from HDAC9 [35, 36]. It remains unknown whether MITR plays a distinct role in osteoclasts. The commercially available *Hdac9* shRNAs we used are predicted to target both the full-length *Hdac9* and *Mitr* transcripts (BLAST alignment and data not shown), and the global *Hdac9*-KO mice have reduced HDAC9 and MITR protein levels [37]. Future work should examine potential roles that are specific to MITR but separate from those of HDAC9.

### Repressors of differentiation, fusion, and activity

Contrary to *Hdac4*, *Hdac9*, and *Mitr*’s early expression, *Hdac5*, *Hdac10*, and *Hdac11* have expression levels that increase after RANKL treatment (Fig 1). Knockdown of any of these three HDACs results in increased osteoclast size and percent demineralization with little to no change in the number of osteoclasts (Figs 3, 6 and 7). Supporting this, knockdown of each HDAC increased *Dc-stamp* expression, which would lead to more fusion and, consequently, larger cells. However, knockdown did not affect *c-Fos* expression or cell number, indicating these HDACs mostly affect genes regulating differentiation, fusion, and possibly activity instead of proliferation (Figs 3, 6 and 7). Knockdown of either *Hdac5* or *Hdac10* showed a trend of increased *Nfatc1* expression (Figs 3H and 6H). NFATc1 regulates expression of genes important for cell fusion (*Dc-stamp*) and resorption activity (*Ctsk*), as well as its own autoamplification after it is stably induced during differentiation [38–40]. Therefore, HDAC-mediated inhibition of *Nfatc1* expression could reduce NFATc1 activity, concomitantly decreasing expression of genes that promote differentiation, fusion, and activity of osteoclasts. Indeed, *Hdac5* shRNA treatment showed trends of increased *Nfatc1* and *Dc-stamp* expression with significantly increased *Ctsk* expression (Fig 3). Supporting this, HDAC5 activity promotes deacetylation and, consequently, destabilization of NFATc1 [41]. Generally, class IIa HDACs rely on recruitment of class I HDACs, typically HDAC3, for deacetylation of targets [15]. Interestingly, HDAC10 interacts with all class I and IIa HDACs and could potentially act as an HDAC recruiter for the repression of targets [13]. Future HDAC5 and HDAC10 studies should concentrate on a potential role for their repression of NFATc1 either cooperatively or separately.

Suppression of *Hdac11* resulted in a milder phenotype than either *Hdac5-* or *Hdac10*-shRNA treatment (Fig 7). This is a surprising result to us considering HDAC11 is more closely related to class I than II HDACs [13], and we have previously shown that loss of expression of *Hdac3*, a class I HDAC, inhibits osteoclast differentiation [23]. Distinct from *Hdac5* and *Hdac10*, *Hdac11* knockdown only increased *Dc-stamp* expression (Fig 7I). This potentially means HDAC11 specifically targets the *Dc-stamp* promoter or fusion genes without affecting the transcription of proteins such as NFATc1, which regulate both differentiation and fusion of osteoclasts. HDAC11 may interact with NFATc1 in a protein-protein interaction to regulate NFATc1’s ability to activate targets such as *Dc-stamp*. The mechanism(s) by which HDAC11 inhibits osteoclast differentiation will be explored in future work. HDAC11 has previously been shown to associate with and deacetylate the *IL-10* promoter in bone marrow-derived antigen-presenting cells ([42]. Surprisingly, HDAC6 and HDAC11 physically interact and oppose each other in regarding to *Il-10* expression; both HDAC6 and HDAC11 associate with the *Il-10* promoter, but HDAC6 promotes expression while HDAC11 represses expression [43]. With the varied expression of different HDACs throughout differentiation, a similar mechanism may exist in osteoclasts. However, as explained in detail below, HDAC6 most likely is not exerting an opposing action on HDAC11 activity in osteoclasts.

### The role of HDAC6

HDAC6 can be recruited to gene promoters to modulate gene expression [43, 44]. Though we successfully reduced *Hdac6* expression, no observable effects on gene expression were seen (Fig 4). Since our four examined genes are far from an exhaustive characterization of altered gene expression by HDAC suppression, it is possible that HDAC6 does have some role in regulating osteoclast-specific gene expression that is outside of our examination or it does so in a redundant manner with another HDAC. Classically, HDAC6 is known to deacetylate α-tubulin, leading to changes in microtubule stabilization and dynamics [45, 46]. It may be that HDAC6’s role in regulating osteoclast differentiation is limited to effecting changes to microtubules but not gene expression. Further characterizing potential gene targets of HDAC6 in osteoclasts is beyond the scope of this work and may be further examined in the future. *In vivo* knockdown of HDAC6 in embryonic stem cells displayed hyperacetylated tubulin [47]. Supporting this, studies in osteoclasts demonstrated that inhibition of HDAC6 activity with chemical inhibitors led to hyperacetylated tubulin and stabilized podosomes [30, 48]. Microtubules are important in helping form and stabilize the podosome belt, which facilitates cell adhesion and matrix resorption by osteoclasts [49]. Knowing this, we predicted knockdown of *Hdac6* to produce a significant effect in the demineralization assay; however, we did not observe any phenotype in terms of osteoclast size, number, or activity after *Hdac6* depletion by shRNA (Fig 4). Though these results are surprising, they are not completely unexpected considering the conflicting results between HDAC6 knockdown and inhibition of activity in other cell types. Importantly, chemical inhibition of HDAC6 led to hyperacetylated tubulin and reduced microtubule dynamics, while siRNA-mediated knockdown of *Hdac6* also showed hyperacetylated tubulin but did not affect microtubule dynamics [50, 51]. In this way, we would not expect *Hdac6* knockdown to impact osteoclast size, number, or activity. Future HDAC6 research should concentrate on additional transcriptional regulatory roles or potential redundancy with Sirtuin 2, a class III HDAC known to also deacetylate α-tubulin [52].

**Fig 8:**
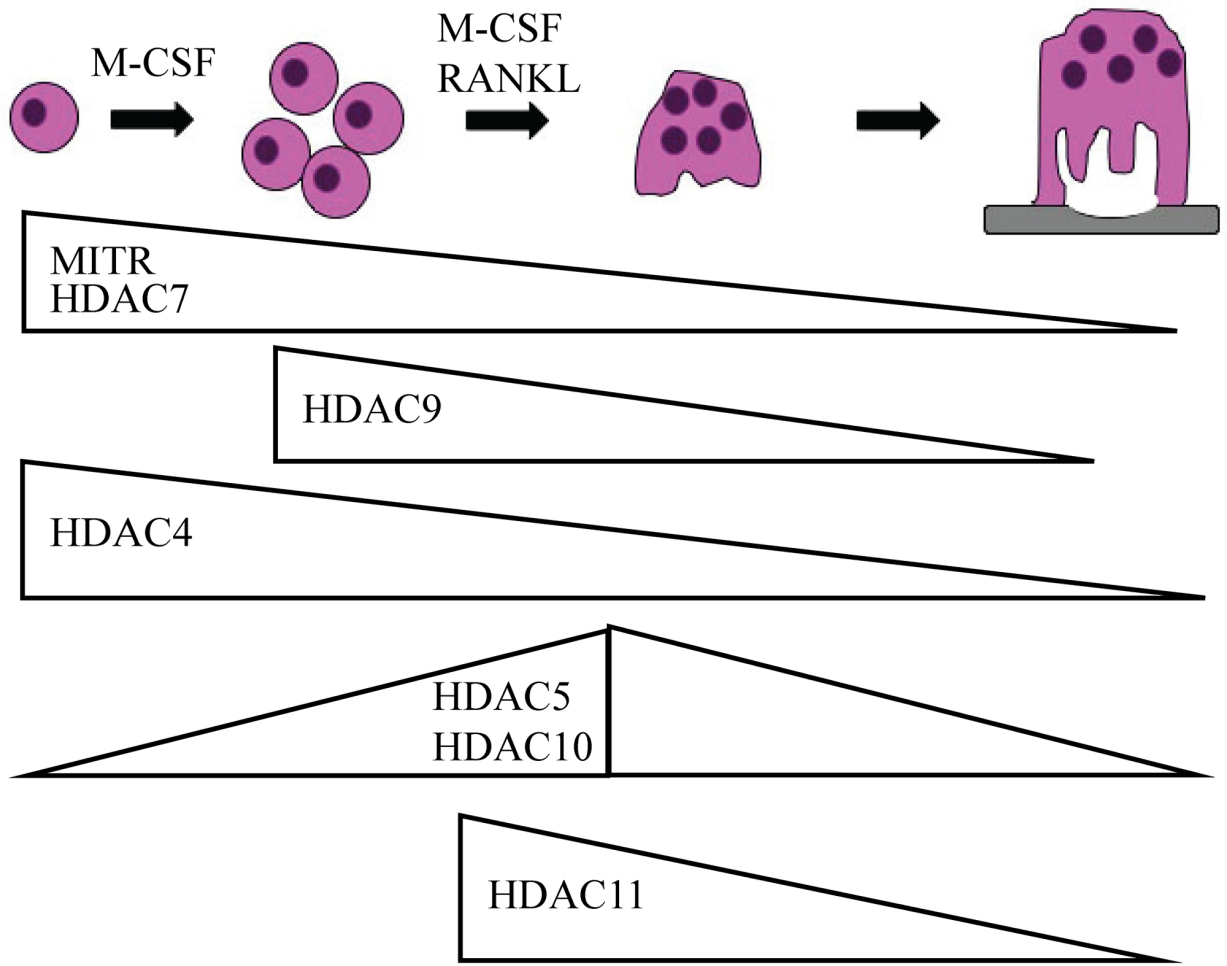
Expression pattern of class II and IV HDACs during osteoclast differentiation. Illustration of the stages of osteoclast differentiation with HDAC expression as determined by the western blots presented in Fig 1.

Overall, we have demonstrated for the first time the results of specifically knocking down *Hdac4*, *5*, *6*, *10*, and *11* in osteoclasts. We found that HDACs differ in their temporal expression pattern during osteoclast differentiation (Fig 8). Except for HDAC6, our studies indicate that class II and IV HDACs have non-redundant roles during osteoclast differentiation. This study serves as an important starting block from which future work examining individual HDACs in osteoclasts should benefit.

## Supporting information

S1 Table Sequence of primers used for qRT-PCR. (DOCX)

S1 Fig Expression of class II and IV *Hdacs* during osteoclast differentiation. BMMs were cultured in M-CSF only (day zero) or in M-CSF and RANKL (day one—day four) to stimulate osteoclast differentiation. qRT-PCR was performed to measure mRNA expression of *Hdacs* during osteoclast differentiation. Graphed data is the mean ± SD from three independent experiments. * p < 0.05; *** p <0.001; ns = not significant compared to day zero.
(TIF)

S2 Fig Full length western blots shown in Fig 1B demonstrating HDAC expression during osteoclast differentiation. The western blots for HDAC10 and HDAC11 are labeled in the following order: 1) Bio-Rad Precision Plus Dual Color Protein Ladder 2) 293T protein lysates overexpressing HDAC 3) day zero 4) day one 5) day two 6) day three 7) day four osteoclast lysates. For HDAC5 full-length blot lanes are 2) 293T protein lysates overexpressing HDAC5 3) day zero 4) day one 5) day two 6) day three 7) day four osteoclast lysates. For HDAC4, HDAC7, HDAC9 and MITR lanes labeled are 3) day zero 4) day one 5) day two 6) day three 7) day four osteoclast lysates. Western blots analyzed for HDAC4 expression have two sets of osteoclast lysates on the blot, and those for HDAC9 and MITR expression have three different sets of lysates.
(TIF)

S3 Fig Suppression of *Hdac4* increased demineralization. qPCR (A) of control and *Hdac4* expressing cells and (B) full length western blot shown in Fig 2F. Number of pits (C) and average area of pits (D) of BMMs that were transduced with control or *Hdac4* shRNAs (*Hdac4*#1 and *Hdac4*#2) and cultured on calcium phosphate-coated plates in the presence of M-CSF and RANKL. * p < 0.05; ** p <0.01, ns = not significant compared to control infected cells.
(TIF)

S4 Fig Suppression of *Hdac5* increases demineralization. qPCR (A) of control and *Hdac5* expressing cells and (B) full length western blot shown in Fig 3F. Number of pits (C) and average area of pits (D) made by BMMs that were infected with control or *Hdac5* shRNAs (*Hdac5*#1 and *Hdac5*#2) and cultured on calcium phosphate-coated plates in the presence of M-CSF and RANKL. * p < 0.05; *** p <0.001 compared to control infected cells.
(TIF)

S5 Fig *Hdac6* suppression has little effect on osteoclast activity. Number of pits (A) and average area of pits (B) of BMMs that were infected with control or *Hdac6* shRNAs (*Hdac6*#1 and *Hdac6*#2) and cultured on calcium phosphate-coated plates in the presence of M-CSF and RANKL. ** p <0.01, ns = not significant compared to control infected cells.
(TIF)

S6 Fig HDAC9 represses osteoclast activity. qPCR (A) of control and *Hdac9* infected cells and (B) full length western blot shown in Fig 5F. Number of pits (C) and average area (D) of pits of BMMs that were infected with control or *Hdac9* shRNAs (*Hdac9*#1 and *Hdac9*#2) and plated on calcium phosphate-coated plates in the presence of M-CSF and RANKL. *** p <0.001, ns = not significant compared to control infected cells.
(TIF)

S7 Fig *Hdac10* knockdown increases demineralization activity of osteoclasts. Number of pits (A) and average area of pits (B) made by BMMs infected with control or *Hdac10* shRNAs (*Hdac10*#1 and *Hdac10*#2) and plated on calcium phosphate-coated plates in the presence of M-CSF and RANKL. * p < 0.05; ns = not significant compared to control infected cells.
(TIF)

S8 Fig *Hdac11* repression enhances osteoclast activity. qPCR (A) of control and *Hdac11* infected cells and (B) full length western blot shown in Fig 7F. Number of pits (C) and average area of pits (D) of BMMs that were infected with control or *Hdac11* shRNAs (*Hdac11*#1 and *Hdac11*#2) and plated on calcium phosphate-coated plates in the presence of M-CSF and RANKL.** p <0.01, *** p <0.001, ns = not significant compared to control infected cells.
(TIF)
