# Supplementary material for: Class II and IV HDACs function as inhibitors of osteoclast differentiation
Source: PLoS One. 2017 Sep 27;12(9):e0185441. doi: 10.1371/journal.pone.0185441 (PMC5617211; doi:10.1371/journal.pone.0185441)
Supplement: S1 Table — (DOCX) [file pone.0185441.s001.docx]

**S1 Table. Sequence of primers used for qRT-PCR**

| **Gene** | **Primers** | **Sequence**  **5’→3’** |
| --- | --- | --- |
| *Cathepsin K (Ctsk)* | Forward | AGGGAAGCAAGCACTGGATA |
| *Cathepsin K (Ctsk)* | Reverse | GCTGGCTGGAATCACATCTT |
| *c-Fos* | Forward | CCAAGCGGAGACAGATCAACTT |
| *c-Fos* | Reverse | TCCAGTTTTTCCTTCTCTTTCAGCAGA |
| *Dc-stamp* | Forward | CAGACTCCCAAATGCTGGAT |
| *Dc-stamp* | Reverse | CTTGTGGAGGAACCTAAGCG |
| *Nfatc1* | Forward | TCATCCTGTCCAACACCAAA |
| *Nfatc1* | Reverse | TCACCCTGGTGTTCTTCCTC |
| *Hdac4* | Forward | CATGGGTACTGCTGTAGGGG |
| *Hdac4* | Reverse | ATGAGCTCCCAAAGCCATC |
| *Hdac5* | Forward | CTGTCCCGTCCGTCTGTCTG |
| *Hdac5* | Reverse | ATGCCATCTGCCGACTCGTT |
| *Hdac6* | Forward | GGAGACAACCCAGTACATGAATGAA |
| *Hdac6* | Reverse | CGGAGGACAGAGCCTGTAG |
| *Hdac9* | Forward | CCAAGTCACTGGGGCATCTT |
| *Hdac9* | Reverse | TGTTCCTCTCCCAGGGTTCT |
| *Hdac10* | Forward | GGCATCGCTGAATGAGTACA |
| *Hdac10* | Reverse | GGATGAGGATCTTGCCACAC |
| *Hdac11* | Forward | GGGGGATCTCAGTGATGGTA |
| *Hdac11* | Reverse | AAGAGAAGCTGCTGTCCGAT |
| *Hprt* | Forward | GAGGAGTCCTGTTGATGTTGCCAG |
| *Hprt* | Reverse | GGCTGGCCTATAGGCTCATAGTGC |
