# Supplementary figures and images for: Class II and IV HDACs function as inhibitors of osteoclast differentiation
Source: PLoS One. 2017 Sep 27;12(9):e0185441. doi: 10.1371/journal.pone.0185441 (PMC5617211; doi:10.1371/journal.pone.0185441)

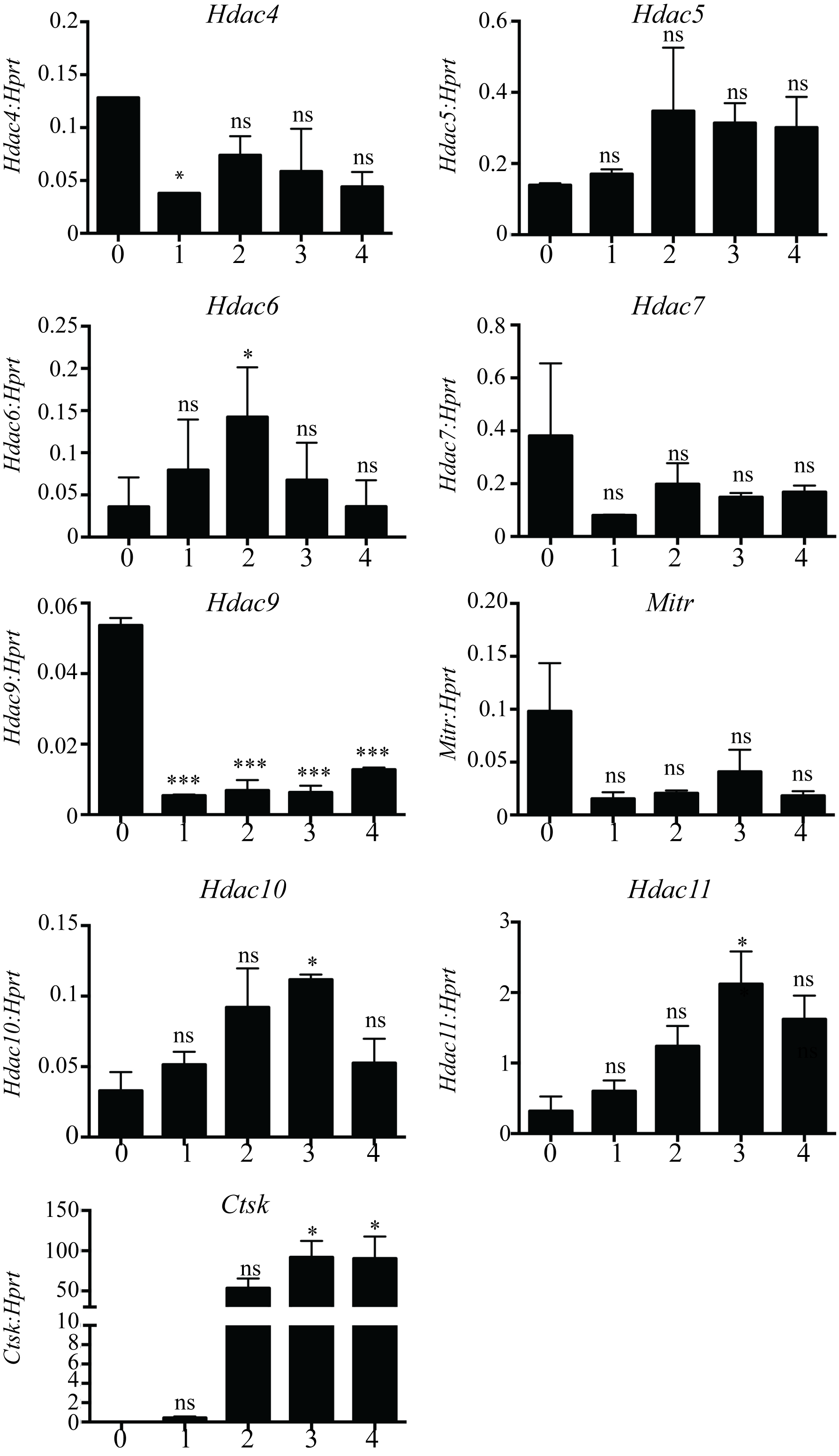

Supplement: S1 Fig — BMMs were cultured in M-CSF only (day zero) or in M-CSF and RANKL (day one—day four) to stimulate osteoclast differentiation. qRT-PCR was performed to measure mRNA expression of Hdacs during osteoclast differentiation. Graphed data is the mean ± SD from three independent experiments. * p < 0.05; *** p <0.001; ns = not significant compared to day zero. (TIF) [file pone.0185441.s002.tif]

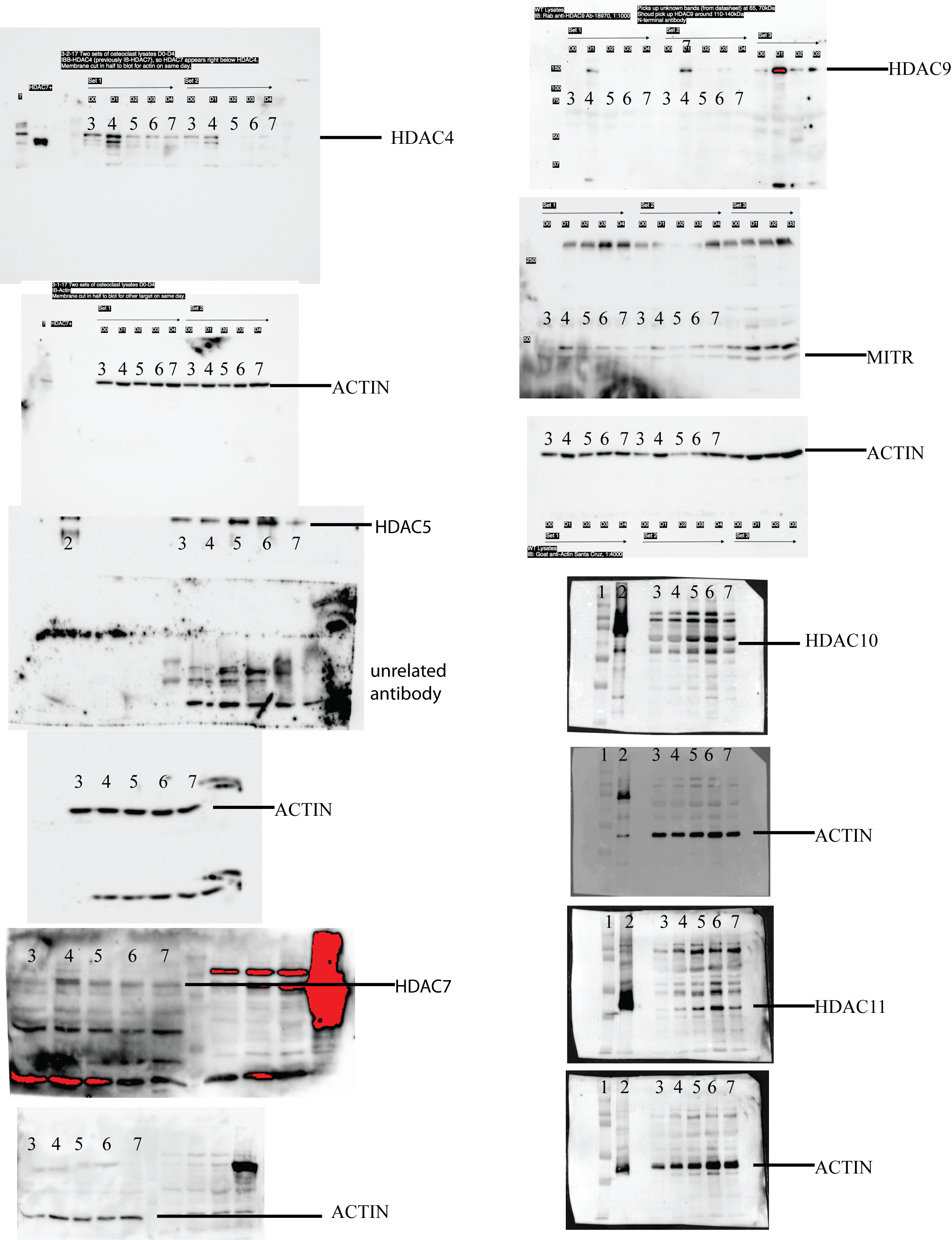

Supplement: S2 Fig — The western blots for HDAC10 and HDAC11 are labeled in the following order: 1) Bio-Rad Precision Plus Dual Color Protein Ladder 2) 293T protein lysates overexpressing HDAC 3) day zero 4) day one 5) day two 6) day three 7) day four osteoclast lysates. For HDAC5 full-length blot lanes are 2) 293T protein lysates overexpressing HDAC5 3) day zero 4) day one 5) day two 6) day three 7) day four osteoclast lysates. For HDAC4, HDAC7, HDAC9 and MITR lanes labeled are 3) day zero 4) day one 5) day two 6) day three 7) day four osteoclast lysates. Western blots analyzed for HDAC4 expression have two sets of osteoclast lysates on the blot, and those for HDAC9 and MITR expression have three different sets of lysates. (TIF) [file pone.0185441.s003.tif]

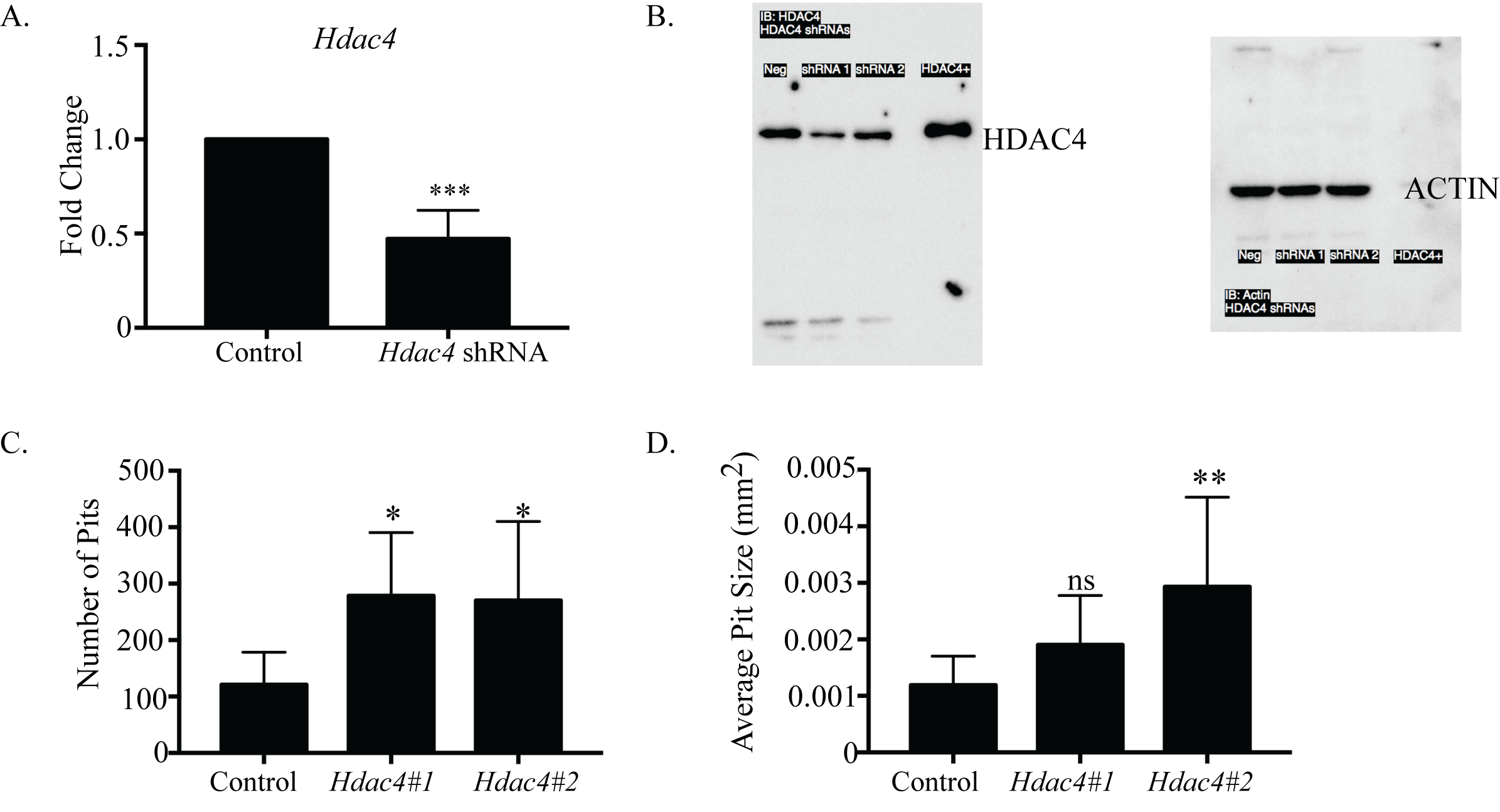

Supplement: S3 Fig — qPCR (A) of control and Hdac4 expressing cells and (B) full length western blot shown in Fig 2F. Number of pits (C) and average area of pits (D) of BMMs that were transduced with control or Hdac4 shRNAs (Hdac4#1 and Hdac4#2) and cultured on calcium phosphate-coated plates in the presence of M-CSF and RANKL. * p < 0.05; ** p <0.01, ns = not significant compared to control infected cells. (TIF) [file pone.0185441.s004.tif]

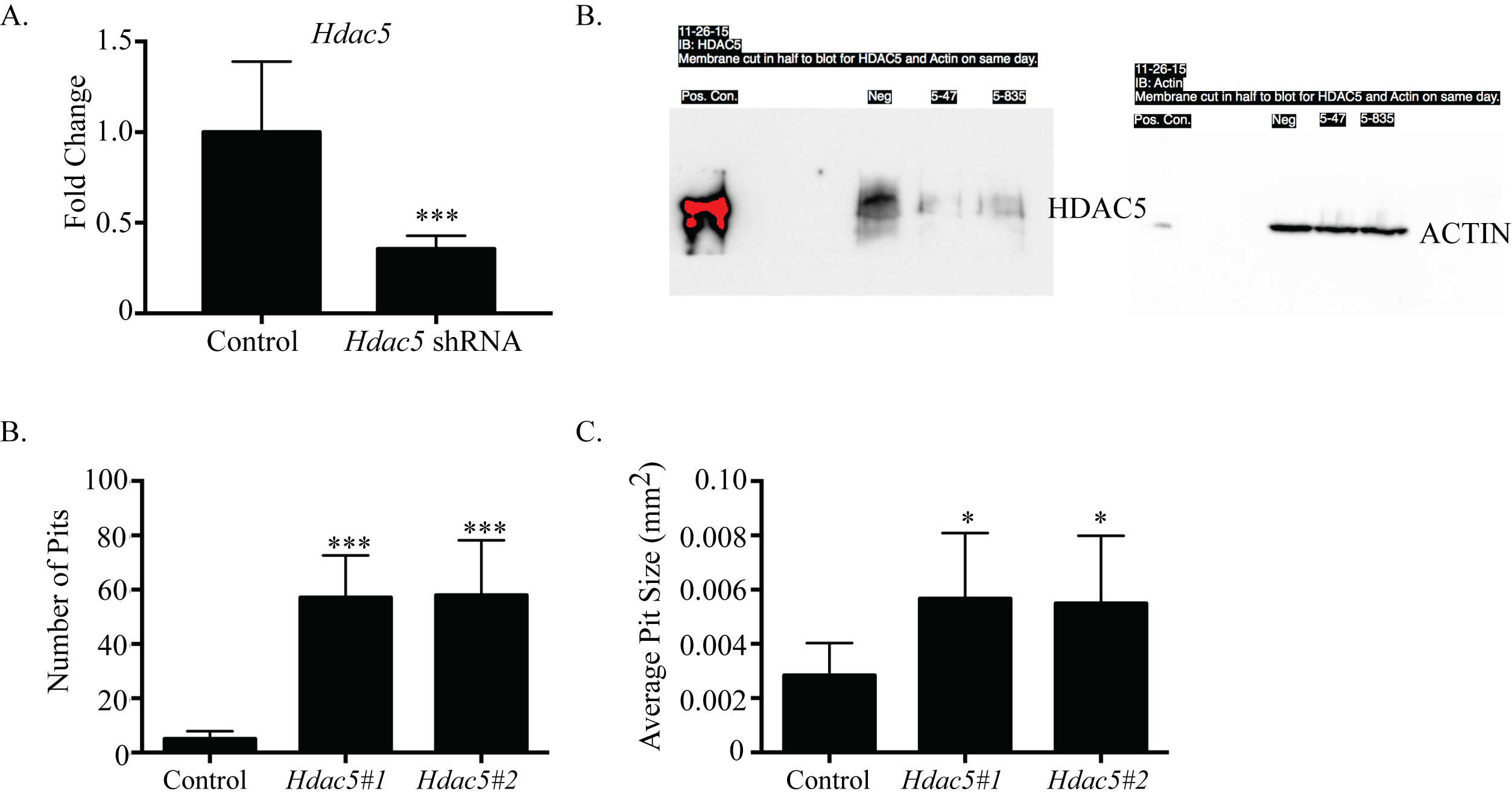

Supplement: S4 Fig — qPCR (A) of control and Hdac5 expressing cells and (B) full length western blot shown in Fig 3F. Number of pits (C) and average area of pits (D) made by BMMs that were infected with control or Hdac5 shRNAs (Hdac5#1 and Hdac5#2) and cultured on calcium phosphate-coated plates in the presence of M-CSF and RANKL. * p < 0.05; *** p <0.001 compared to control infected cells. (TIF) [file pone.0185441.s005.tif]

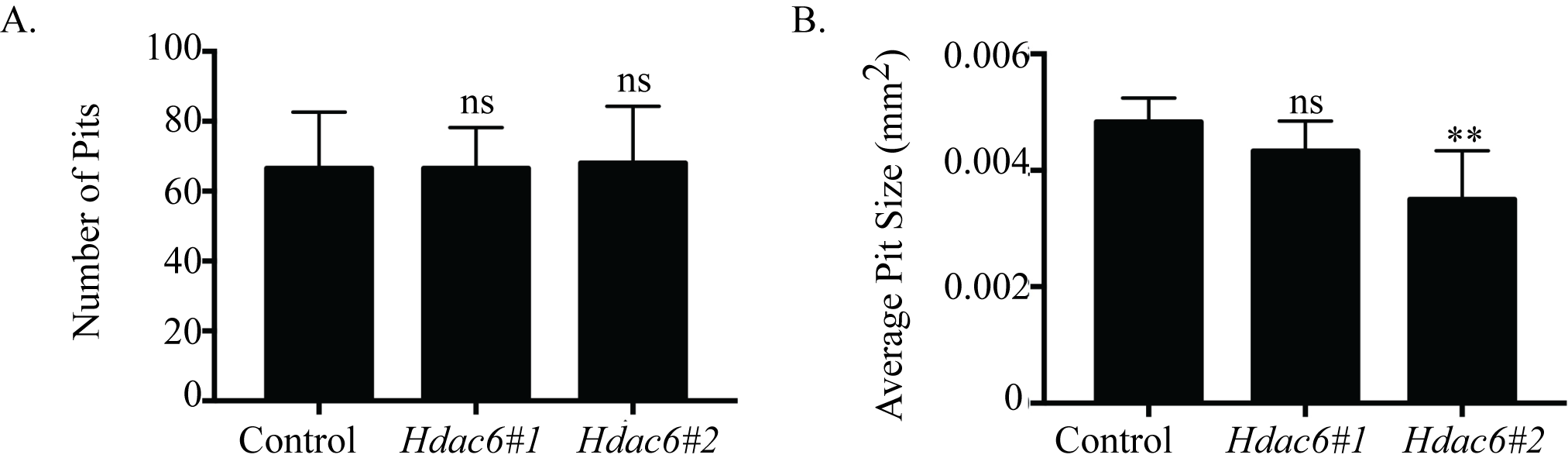

Supplement: S5 Fig — Number of pits (A) and average area of pits (B) of BMMs that were infected with control or Hdac6 shRNAs (Hdac6#1 and Hdac6#2) and cultured on calcium phosphate-coated plates in the presence of M-CSF and RANKL. ** p <0.01, ns = not significant compared to control infected cells. (TIF) [file pone.0185441.s006.tif]

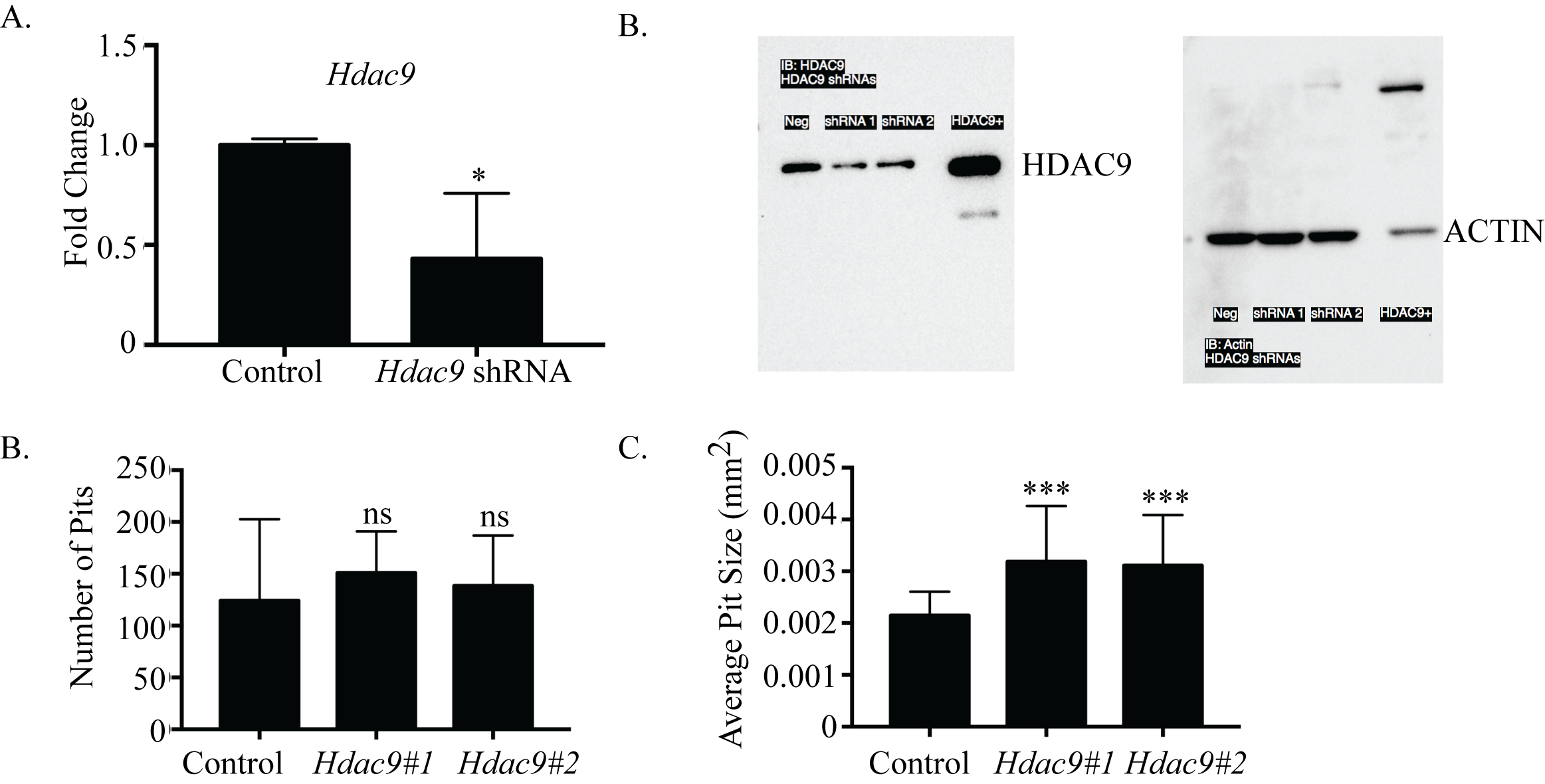

Supplement: S6 Fig — qPCR (A) of control and Hdac9 infected cells and (B) full length western blot shown in Fig 5F. Number of pits (C) and average area (D) of pits of BMMs that were infected with control or Hdac9 shRNAs (Hdac9#1 and Hdac9#2) and plated on calcium phosphate-coated plates in the presence of M-CSF and RANKL. *** p <0.001, ns = not significant compared to control infected cells. (TIF) [file pone.0185441.s007.tif]

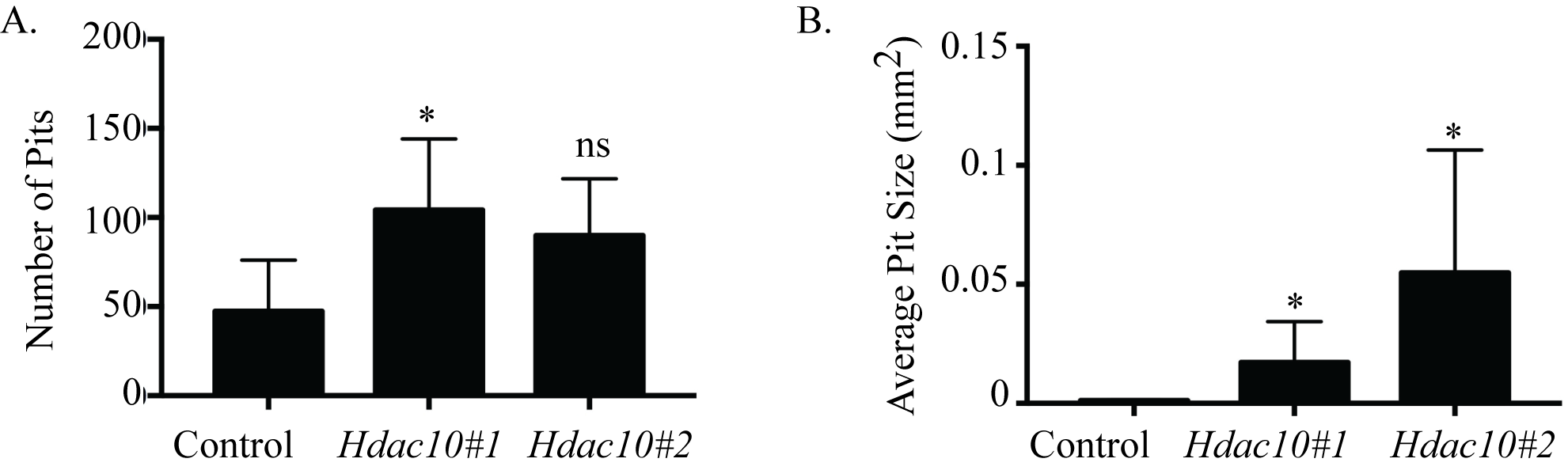

Supplement: S7 Fig — Number of pits (A) and average area of pits (B) made by BMMs infected with control or Hdac10 shRNAs (Hdac10#1 and Hdac10#2) and plated on calcium phosphate-coated plates in the presence of M-CSF and RANKL. * p < 0.05; ns = not significant compared to control infected cells. (TIF) [file pone.0185441.s008.tif]

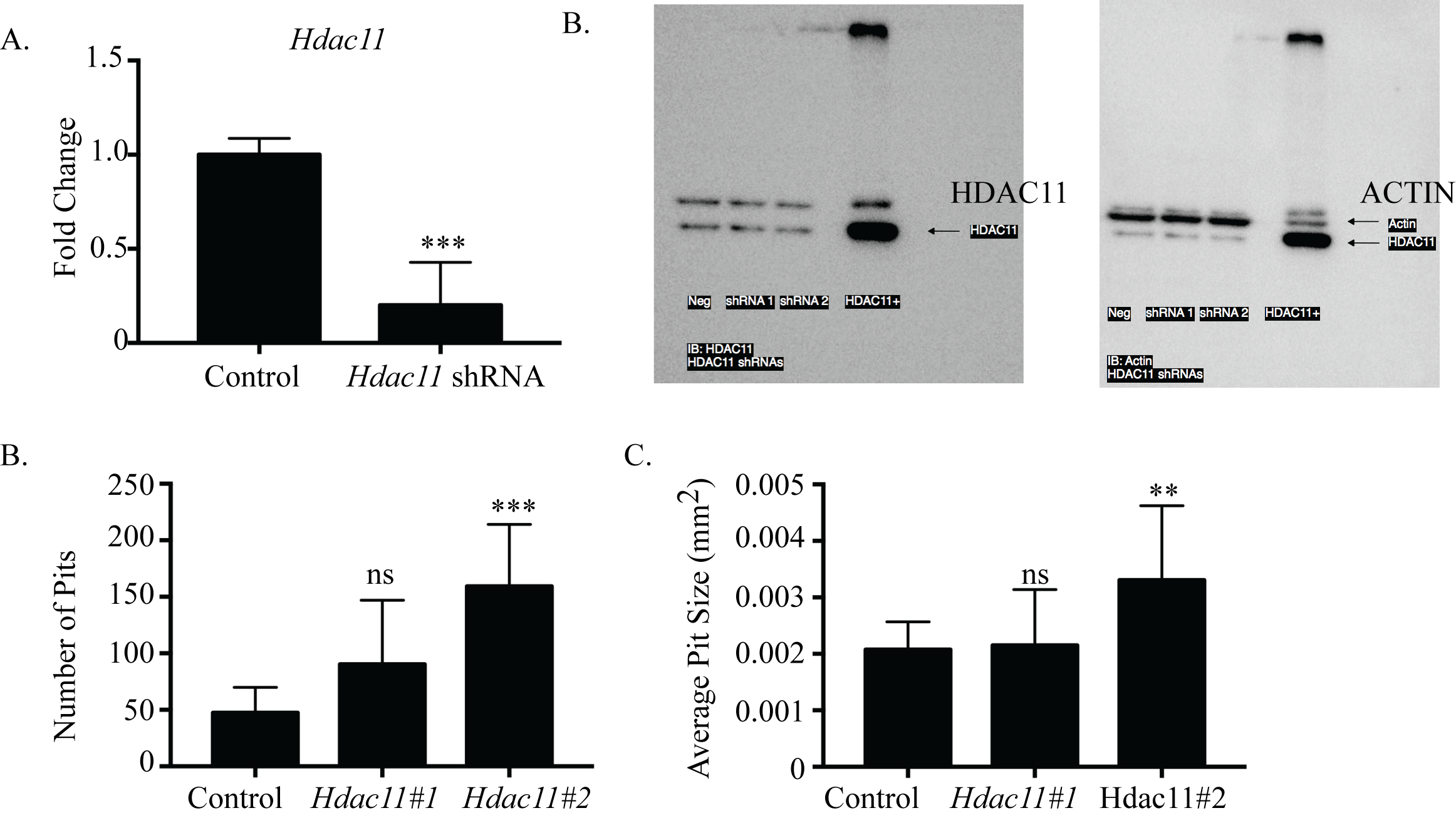

Supplement: S8 Fig — qPCR (A) of control and Hdac11 infected cells and (B) full length western blot shown in Fig 7F. Number of pits (C) and average area of pits (D) of BMMs that were infected with control or Hdac11 shRNAs (Hdac11#1 and Hdac11#2) and plated on calcium phosphate-coated plates in the presence of M-CSF and RANKL.** p <0.01, *** p <0.001, ns = not significant compared to control infected cells. (TIF) [file pone.0185441.s009.tif]
